# Supplementary material for: Dietary Folic Acid Supplementation Attenuates Maternal High-Fat Diet-Induced Fetal Intrauterine Growth Retarded via Ameliorating Placental Inflammation and Oxidative Stress in Rats
Source: Nutrients. 2023 Jul 24;15(14):3263. doi: 10.3390/nu15143263 (PMC10385450; doi:10.3390/nu15143263)
Supplement: Supplementary file 1 [file nutrients-15-03263-s001.zip › nutrients-2479595-supplementary.pdf]

Table S1. Composition of the diets

| Diet component | CON | HFD | FA | HFD+FA |
|---|---|---|---|---|
| Casein (g/kg) | 189.57 | 258.46 | 189.57 | 258.46 |
| Corn starch (g/kg) | 479.81 | n/a | 479.81 | n/a |
| Maltodextrin (g/kg) | 118.48 | 161.54 | 118.48 | 161.54 |
| Sucrose (g/kg) | 65.21 | 88.91 | 65.21 | 88.91 |
| Cellulose (g/kg) | 47.39 | 64.62 | 47.39 | 64.62 |
| Pork lard (g/kg) | 18.96 | 316.62 | 18.96 | 316.62 |
| Soya bean oil (g/kg) | 23.70 | 32.31 | 23.70 | 32.31 |
| Cystine (g/kg) | 2.84 | 3.88 | 2.84 | 3.88 |
| Mineral mix (g/kg) | 9.48 | 12.92 | 9.48 | 12.92 |
| Vitamin mix without folic acid (g/kg) | 9.48 | 12.92 | 9.48 | 12.92 |
| Choline (g/kg) | 1.90 | 2.58 | 1.90 | 2.58 |
| Calcium carbonate (g/kg) | 5.215.21 | 7.117.11 | 5.215.21 | 7.117.11 |
| Dicalcium phosphate (g/kg) | 12.32 | 16.80 | 12.32 | 16.80 |
| Potassium citrate (g/kg) | 15.64 | 21.32 | 15.64 | 21.32 |
| Folic acid (mg/kg) | 2 | 2 | 5 | 5 |

CON, normal fat with normal folic acid diet; HFD, high fat with normal folic acid diet; FA, normal fat with folic acid supplementation diet; HFD+FA, high fat with folic acid supplementation diet; n/a, not applicable.

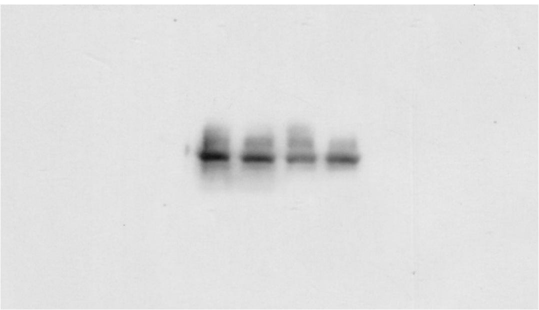

**Figure S1: SIRT1 in the placenta of Western blot.** The molecular mass was 120KD. The sample loading sequence was the CON group, FA group, HFD group and HFD+FA group from left to right.

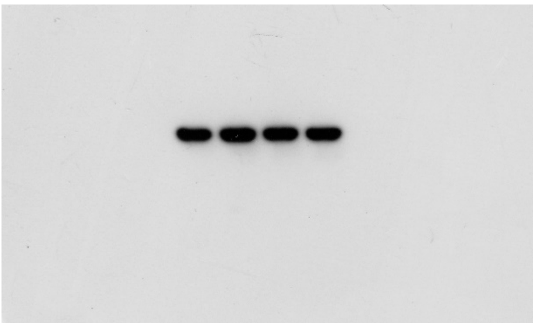

**Figure S2: β-actin in the placenta of Western blot.** The molecular mass was 45KD. The sample loading sequence was the CON group, FA group, HFD group and HFD+FA group from left to right.

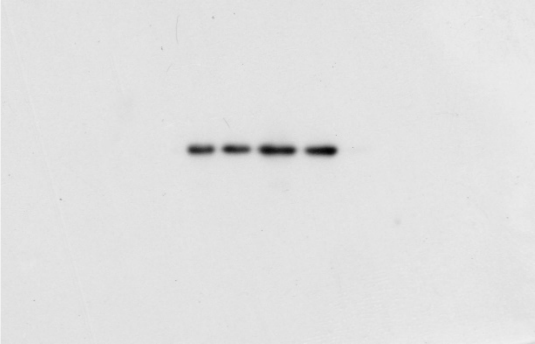

**Figure S3: Nucl p65 in the placenta of Western blot.** The molecular mass was 65KD. The sample loading sequence was the CON group, FA group, HFD group and HFD+FA group from left to right.

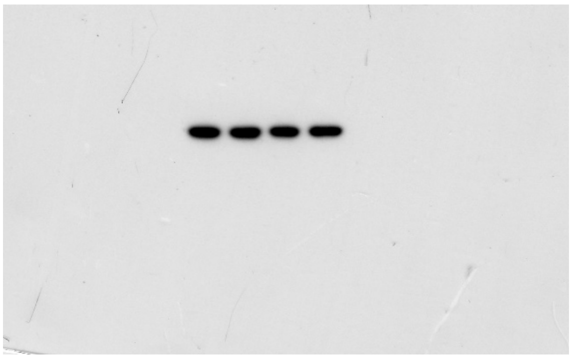

**Figure S4: Lamin B in the placenta of Western blot.** The molecular mass was 68KD. The sample loading sequence was the CON group, FA group, HFD group and HFD+FA group from left to right.

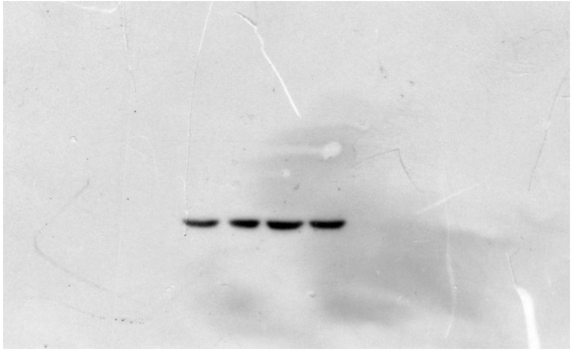

**Figure S5: Cyto p65 in the placenta of Western blot.** The molecular mass was 65KD. The sample loading sequence was the CON group, FA group, HFD group and HFD+FA group from left to right.

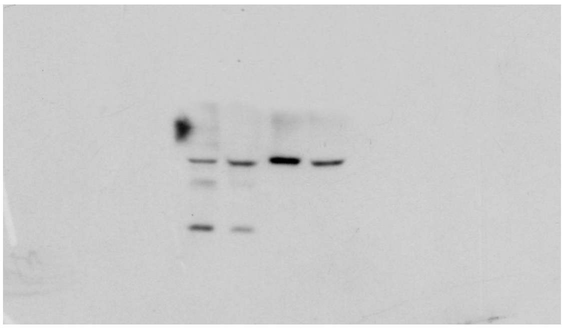

**Figure S6: p-IκBα in the placenta of Western blot.** The molecular mass was 40KD. The sample loading sequence was the CON group, FA group, HFD group and HFD+FA group from left to right.

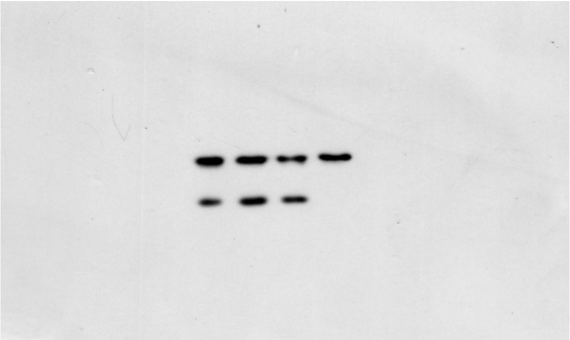

**Figure S7: IκBα in the placenta of Western blot.** The molecular mass was 39KD. The sample loading sequence was the CON group, FA group, HFD group and HFD+FA group from left to right.

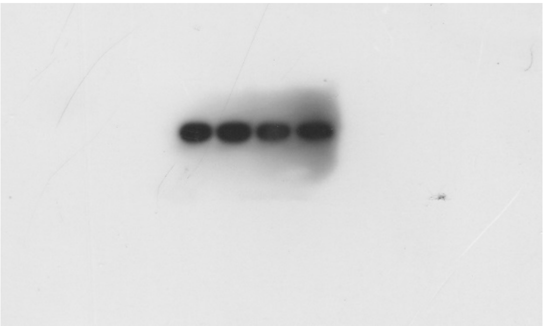

**Figure S8: β-actin in the placenta of Western blot.** The molecular mass was 45KD. The sample loading sequence was the CON group, FA group, HFD group and HFD+FA group from left to right.

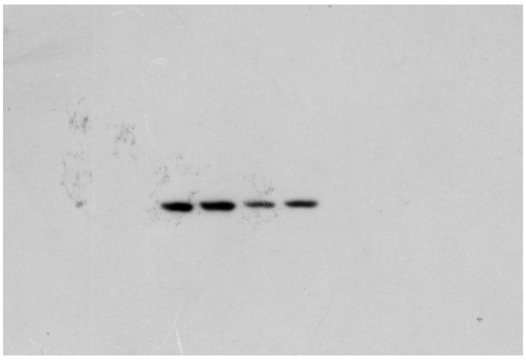

**Figure S9: Nucl Nrf2 in the placenta of Western blot.** The molecular mass was 100KD. The sample loading sequence was the CON group, FA group, HFD group and HFD+FA group from left to right.

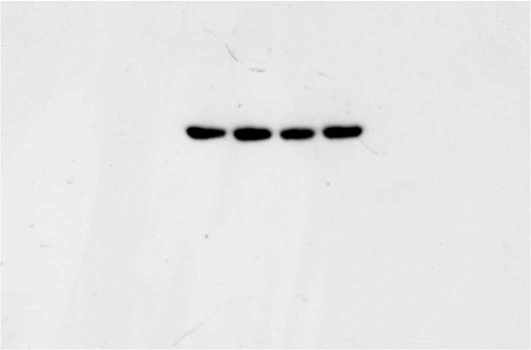

**Figure S10: Lamin B in the placenta of Western blot.** The molecular mass was 68KD. The sample loading sequence was the CON group, FA group, HFD group and HFD+FA group from left to right.

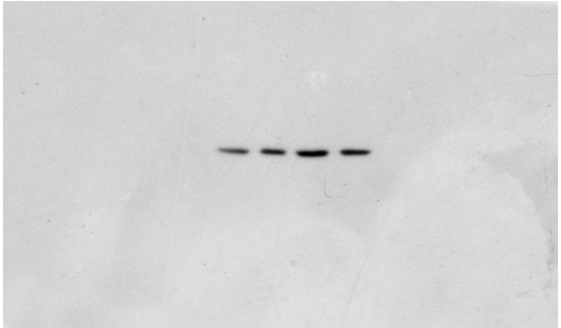

**Figure S11: Cyto Nrf2 in the placenta of Western blot.** The molecular mass was 100KD. The sample loading sequence was the CON group, FA group, HFD group and HFD+FA group from left to right.

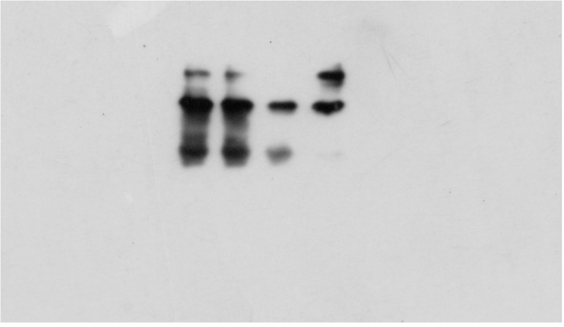

**Figure S12: NQO1 in the placenta of Western blot.** The molecular mass was 29KD. The sample loading sequence was the CON group, FA group, HFD group and HFD+FA group from left to right.

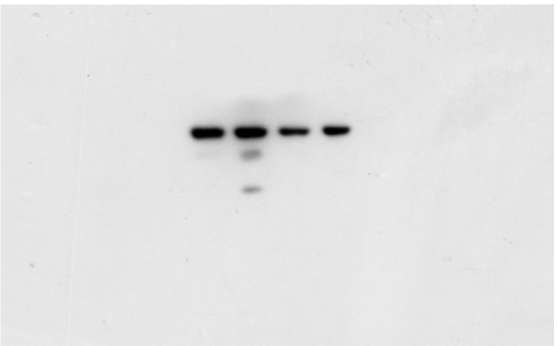

**Figure S13: HO-1 in the placenta of Western blot.** The molecular mass was 28KD. The sample loading sequence was the CON group, FA group, HFD group and HFD+FA group from left to right.

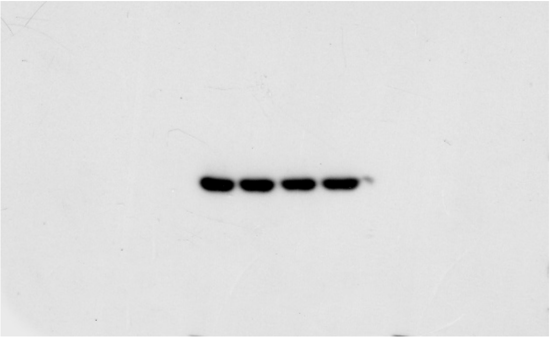

**Figure S14: β-actin in the liver of Western blot.** The molecular mass was 45KD. The sample loading sequence was the CON group, FA group, HFD group and HFD+FA group from left to right.
